# Molecular Characterization of Reduced Susceptibility to Biocides in Clinical Isolates of *Acinetobacter baumannii*

**DOI:** 10.3389/fmicb.2017.01836

**Published:** 2017-09-26

**Authors:** Fei Lin, Ying Xu, Yaowen Chang, Chao Liu, Xu Jia, Baodong Ling

**Affiliations:** ^1^Sichuan Province College Key Laboratory of Structure-Specific Small Molecule Drugs, School of Pharmacy, Chengdu Medical College, Chengdu, Sichuan, China; ^2^Non-coding RNA and Drug Discovery Key Laboratory of Sichuan Province, Chengdu Medical College, Chengdu, Sichuan, China; ^3^Clinical Laboratory, the First Affiliated Hospital, Chengdu Medical College, Chengdu, Sichuan, China

**Keywords:** *Acinetobacter baumannii*, antibiotics, biocides, chlorhexidine, triclosan, efflux pump genes, MLST, PFGE

## Abstract

Active efflux is regarded as a common mechanism for antibiotic and biocide resistance. However, the role of many drug efflux pumps in biocide resistance in *Acinetobacter baumannii* remains unknown. Using biocide-resistant *A. baumannii* clinical isolates, we investigated the incidence of 11 known/putative antimicrobial resistance efflux pump genes (*adeB, adeG, adeJ, adeT1, adeT2, amvA, abeD, abeM, qacE, qacE*Δ*1*, and *aceI*) and triclosan target gene *fabI* through PCR and DNA sequencing. Reverse transcriptase quantitative PCR was conducted to assess the correlation between the efflux pump gene expression and the reduced susceptibility to triclosan or chlorhexidine. The *A. baumannii* isolates displayed high levels of reduced susceptibility to triclosan, chlorhexidine, benzalkonium, hydrogen peroxide, and ethanol. Most tested isolates were resistant to multiple antibiotics. Efflux resistance genes were widely distributed and generally expressed in *A. baumannii*. Although no clear relation was established between efflux pump gene expression and antibiotic resistance or reduced biocide susceptibility, triclosan non-susceptible isolates displayed relatively increased expression of *adeB* and *adeJ* whereas chlorhexidine non-susceptible isolates had increased *abeM* and *fabI* gene expression. Increased expression of *adeJ* and *abeM* was also demonstrated in multiple antibiotic resistant isolates. Exposure of isolates to subinhibitory concentrations of triclosan or chlorhexidine induced gene expression of *adeB, adeG, adeJ* and f*abI*, and *adeB*, respectively. A point mutation in FabI, Gly95Ser, was observed in only one triclosan-resistant isolate. Multiple sequence types with the major clone complex, CC92, were identified in high level triclosan-resistant isolates. Overall, this study showed the high prevalence of antibiotic and biocide resistance as well as the complexity of intertwined resistance mechanisms in clinical isolates of *A. baumannii*, which highlights the importance of antimicrobial stewardship and resistance surveillance in clinics.

## Introduction

*Acinetobacter baumannii* is an important human nosocomial pathogen which causes a variety of infections, including bacteremia, pneumonia, meningitis, septicemia, urinary tract infections, wound and skin infections (Bergogne-Berezin and Towner, 1996; Peleg et al., 2008; La Forgia et al., 2010). *A. baumannii* is a major threat to human health, has the ability to persist and colonize various environments and also displays multiple drug resistance to antimicrobial agents such as antibiotics and biocides (Bergogne-Berezin and Towner, 1996; Babaei et al., 2015; Chang et al., 2015). Consequently, it is challenging to control infection and dissemination of *A. baumannii* in medical settings.

Biocides are commonly used in hospitals, laboratories, industries as well as in households for inhibiting or killing pathogenic bacteria and thus play an important role in reducing the dissemination of pathogenic organisms in hospital environments (McDonnell and Russell, 1999). However, reduced susceptibility to biocides among bacterial species, including *A. baumannii*, has been increasingly observed in patients in recent years (Peleg and Paterson, 2006; Gnanadhas et al., 2013; Babaei et al., 2015; Fernández-Cuenca et al., 2015). To date, several studies have reported that *A. baumannii* clinical isolates have reduced susceptibility to commonly used biocides such as alcohols, chlorhexidine, formaldehyde, glutaraldehyde, hydrogen peroxide, hypochlorite, iodine and iodophors, quaternary ammonium compounds (such as benzalkonium), and triclosan (Wisplinghoff et al., 2007; Chen et al., 2009; Ray et al., 2010; Fernández-Cuenca et al., 2015). Furthermore, cross-resistance between biocide exposure and antibiotic resistance (and vice versa) in bacteria has been recently reported (Curiao et al., 2015; Fernández-Cuenca et al., 2015). However, the molecular characterization of reduced biocide susceptibility and its relationship with antibiotic resistance in *A. baumannii* remains to be fully determined.

Bacteria have the ability to resist antibiotics and biocide exposure by intrinsic or acquired mechanisms, such as phenotypic changes, antimicrobial inactivation, target alterations and drug access prevention (McDonnell and Russell, 1999; Gnanadhas et al., 2013). Of these resistance mechanisms, *A. baumannii* encodes for a large number of drug efflux pumps with broad substrate profiles that contribute toward the high level of intrinsic resistance to structurally-unrelated agents (Fournier et al., 2000; Ling et al., 2016). These resistance determinants facilitate the evolution of acquired resistance (Lin et al., 2009, 2017b; Coyne et al., 2011; Nowak et al., 2015; Ling et al., 2016). Indeed, the drug efflux pumps that are known to mediate intrinsic and/or acquired resistance to conventional antibiotics and biocides include the efflux systems of the resistance-nodulation-cell division (RND) superfamily (AdeABC, AdeFGH, AdeIJK, and AbeD) (Rumbo et al., 2013; Srinivasan et al., 2015; Ling et al., 2016), AmvA pump of the major facilitator superfamily (MFS) (Rajamohan et al., 2010a), AbeM of the multidrug and toxic compound extrusion (MATE) family (Su et al., 2005), QacE and QacEΔ1 of the small drug resistance (SMR) family (Fournier et al., 2000; Taitt et al., 2014; Babaei et al., 2015), and AceI of the proteobacterial antimicrobial compound efflux (PACE) family (Hassan et al., 2013, 2015). Additionally, triclosan, a broad-spectrum biocide, is an inhibitor of the fatty acid biosynthetic enzyme, FabI, which is an enoyl-acyl carrier protein reductase and encoded by *fabI* gene. Mutations within *fabI* contribute to *A. baumannii* resistance to triclosan (Heath et al., 1999; Chen et al., 2009).

Despite the roles of these known resistance determinants, a scientific data gap exists for the simultaneous examination of these resistance mechanisms in clinical isolates of *A. baumannii*. In this study, the susceptibility of *A. baumannii* isolates to antibiotics and biocides collected from the First Affiliated Hospital of Chengdu Medical College, China, was investigated to assess the role of drug resistance transporters in biocide resistance. Our study focuses on the expression of drug efflux genes (including the first assessment of *aceI* expression) in relation to *A. baumannii* susceptibility to triclosan and chlorhexidine. High-level triclosan-resistant isolates were also characterized by molecular typing techniques. The results revealed the role of several efflux pumps in chlorhexidine or triclosan resistance and the complexity of biocide resistance mechanisms, which highlight the importance for antimicrobial stewardship including the use of biocide agents.

## Materials and methods

### Bacterial strains and growth conditions

A total of 47 *A. baumannii* clinical isolates were derived from the blood, sputum or swab samples of patients from different departments at the First Affiliated Hospital of Chengdu Medical College, China from 2014 to 2015. Bacteria were aerobically grown at 37°C in Mueller-Hinton (MH) broth or agar (Oxoid, England) or in Luria-Bertani (LB) broth (Lin et al., 2009). These isolates were identified by ATB New (bioMérieux, France) (Chang et al., 2015) and further verified by PCR products of *rpoB* (Wang et al., 2014) and *16S rRNA* gene (Chiang et al., 2011) with the primers included in Table S1. The PCR products were sequenced by Tsingke Biological Technology (Tsingke, Chengdu, China), followed by sequence alignment using BLAST (http://blast.ncbi.nlm.nih.gov/Blast.cgi).

### Antimicrobial susceptibility testing

Antibiotics and biocides used in this study were purchased commercially. Chlorhexidine acetate and triclosan were purchased from Dalian Meilun Biological Technology (Dalian, China), benzalkonium bromide from Chengdu Dingdangchem Medical Technology (Chengdu, China), hydrogen peroxide, ethanol and sodium hypochlorite from Chengdu Kelong Chemical Reagent Factory (Chengdu, China). Biocide stock solutions of 10 mg/L of chlorhexidine acetate or benzalkonium bromide in deionized water and triclosan in dimethyl sulfoxide were prepared. Susceptibility testing for antibiotics and biocides was carried out using the broth microdilution method in accordance with the guidelines of the Clinical and Laboratory Standards Institute (CLSI) (CLSI, 2014). The ranges of biocide concentrations evaluated were 0.25–256 μg/ml (chlorhexidine acetate), 0.25–256 μg/ml (benzalkonium bromide), 0.25–256 μg/ml (triclosan), 1.25–2,560 μg/ml (sodium hypochlorite), 2.9–6,021 mM (hydrogen peroxide), and 7.5–75% (vol/vol) (ethanol). The minimal inhibitory concentration (MIC) was defined as the lowest concentration of an antimicrobial agent at which no bacterial growth was observed visually after incubation at 37°C for 18 h (CLSI, 2015).

### PCR amplification and sequencing

Eleven known drug resistance transporter genes *abeD* (Srinivasan et al., 2015), *aceI* (Hassan et al., 2013), *adeB, adeG, adeJ* (Coyne et al., 2011), *adeT1, adeT2* (Srinivasan et al., 2011), *abeM* (Su et al., 2005), *amvA* (Rajamohan et al., 2010a), *qacE, qacE*Δ*1* (Mahzounieh et al., 2014; Babaei et al., 2015), and the triclosan target gene, *fabI* (Rajamohan et al., 2010b; Fernando et al., 2014), were PCR amplified to determine their distribution in the collected clinical isolates. The primers used are listed in Table S1. PCR amplification of the target genes was performed in a 50 μl volume containing 0.2 mM of each deoxy nucleotide, 0.5 μM of each primer, 1.25 U of Taq polymerase, and 5 μl of 10 × buffer (Vazyme, China). The reaction was amplified using a program consisting of 94°C for 5 min followed by 30 cycles of 94°C for 30 or 60 s, annealing at 50–55°C for 30 or 50 s and extension at 72°C for 30–90 s, and a final extension at 72°C for 10 min. The PCR products were verified by DNA sequencing.

### Multilocus sequence typing (MLST)

MLST was used to characterize the seven housekeeping genes: *gltA* (encoding citrate synthase), *gyrB* (DNA gyrase subunit B), *gdhB* (glucoside hydrogenase B), *recA* (homologous recombination factor), *cpn60* (60-kDa chaperonin), *gpi* (glucose-6-phosphate isomerase) and *rpoD* (RNA polymerase sigma factor) (http://pubmlst.org/abaumannii/info/primers-Oxford.shtml). Amplification reactions were carried out as described previously (Woo et al., 2011). eBURST (Version3, http://eburst.mlst.net/) was used to compare the *A. baumannii* clinical isolate sequence types (ST) to the existing STs and to assign isolates to their clonal complexes. A clonal complex (CC) is defined as a set of similar ST(s) having the same alleles at ≥6 of 7 loci (Aanensen and Spratt, 2005).

### Pulsed-field gel electrophoresis (PFGE)

PFGE was carried out as previously described (Seifert et al., 2005). *A. baumannii* isolates were grown on MH plates overnight at 37°C. Strains were lysed with proteinase K (20 mg/ml stock solution). The plugs were thoroughly washed and then digested using *Apa*I restriction enzyme (Takara, China). Restricted DNA fragments were separated using CHEF Mapper XA system (Bio-Rad, Hercules, CA, USA). The gels were run at 14°C in 0.5 × TBE buffer for 18 h, with pulse time of 5–35 s. *Xba*I-digested *Salmonella enterica* ser. Braenderup H9812 was used as the molecular marker (Dhanoa et al., 2015). Data analysis was based on the Dice coefficient of similarity analyzed by the BioNumerics 3.0 software (Applied Maths, Belgium) using the Unweighted Pair Group Method with Arithmetic Averages (UPGMA) algorithm at 1.5% band position tolerance. Pulsotype designation was based on isolates showing ≥80% relatedness.

### Reverse transcriptase quantitative PCR (qRT-PCR) analysis

Overnight cultures of bacterial strains were subcultured in LB (1:100 dilution) and allowed to grow to an OD_600_ of 0.6–0.8. *A. baumannii* cells were exposed to a subinhibitory concentration (1/2 MIC of triclosan or chlorhexidine; see results for the MIC values of chlorhexidine and triclosan) and grown in parallel with control (no biocide addition) for 2 h. One milliliter of the cell culture was centrifuged and cells were collected for total RNA extraction using TRIzol reagent (Invitrogen). To remove contaminating genomic DNA, the samples were treated with RNase free DNase I based on the manufacturer's instructions (Takara). qRT-PCR analysis was carried out for three RND pump-encoding genes (*adeB, adeG*, and *adeJ*), the MATE family pump gene *abeM*, the *Acinetobacter* chlorhexidine efflux protein gene *aceI*, and the triclosan target-encoding gene *fabI*. The primers used for qRT-PCR analysis are listed in Table S1. Reverse transcription was performed by using the M-MLV Reverse Transcriptase (Invitrogen). The expression of genes was determined by quantitative PCR using SsoFast EvaGreen Supermix (Bio-Rad) with the following cycle profile: 1 cycle at 95°C for 3 min, followed by 40 cycles at 95°C for 15 s, 55°C for 15 s. Relative expression was determined using the cycle threshold (ΔΔ*Ct*) method on the Bio-Rad CFX96 real-time system (Bio-Rad). All reactions were carried out in triplicate with at least two biological replicates. The relative expression levels of target genes were normalized to those of the 16S rRNA gene (Arroyo et al., 2011; Fernando and Kumar, 2012; Kuo et al., 2012; Lin et al., 2017a,b). Data analysis was carried out according to the manufacturer's instructions using the Bio-Rad CFX 3.0 software which is designed for Bio-Rad CFX real-time PCR detection system. Expression data is presented by the mean plus/minus one standard deviation.

## Results

### Clinical information of the isolates

*A. baumannii* clinical isolates (*n* = 47) were collected from three different locations including sputum (*n* = 42), throat swabs (*n* = 4) and blood (*n* = 1) from patients at the First Affiliated Hospital of Chengdu Medical College. Epidemiological analysis of the 47 patients (30 males and 17 females) revealed that 22 were ≥70 years old and 15 were between 60 and 70 years old. Overall, these two patient cohorts account for 78.81% (37/47) of the total isolates. Among the isolates, 22 were collected from intensive care units and 9 were collected from the respiratory department. Most of the clinical specimens were sputum, which is consistent with *A. baumannii* as a major pathogen associated with respiratory tract infection (Bergogne-Berezin and Towner, 1996; Babaei et al., 2015). The clinical characteristics of the 47 patients with *A. baumannii* infection are summarized in Table 1. Based upon previous antimicrobial resistance and treatment concerns for *A. baumannii*, we decided to investigate the susceptibility to antibiotics and biocides in *A. baumannii* and underlying resistance mechanisms.

**Table 1:** Clinical characteristics of 47 patient *A. baumannii* isolates used in this study.

**Gender**	**Total number [*n* (%)]**	**Source**	**Total number [*n* (%)]**	**Department**	**Total number [*n* (%)]**	**Age (years)**	**Total number [*n* (%)]**
Male	30 (63.83%)	Blood	1 (2.13%)	Intensive care unit	22 (46.81%)	21–60	10 (21.28%)
Female	17 (36.17%)	Sputum	42 (89.36%)	Respiratory	9 (19.15%)	60–70	15 (31.91%)
		Swab	4 (8.51%)	Neurosurgery	6 (12.77%)	>70	22 (46.81%)
				Infectious diseases department	1 (2.13%)		
				Other wards	9 (19.15%)		

### Susceptibility of *A. baumannii* isolates to antibiotics and biocides

The results of antibiotic and biocide susceptibility testing are shown in Table 2 and Tables S2, S3. Together, 15 antibiotics and 6 biocides were tested. Based on the interpretative categories from the CLSI (CLSI, 2015), the tested isolates were frequently multidrug resistant (64% [30/47]), including resistance to β-lactams, aminoglycosides, fluoroquinolones and tetracyclines (Table 2 and Table S2). In particular, the rates of resistance to carbapenems and third-generation cephalosporins, amikacin, and minocycline were, respectively, about 60, 40, and 34%.

**Table 2:** Antimicrobial susceptibility of the clinical isolates of *A. baumannii* (*n* = 47)^a^.

**Antimicrobial agents**	**MIC (μg/mL)**			**S (%)**	**I (%)**	**R (%)**
	**MIC range**	**MIC_50_**	**MIC_90_**			
**ANTIBIOTICS**						
Ceftazidime	16–1,024	128	256	0	36.17	63.83
Cefotaxime	4–256	64	128	31.91	2.13	65.96
Ceftriaxone	8–512	128	256	17.02	19.15	63.83
Imipenem	0.0625–64	16	64	36.17	2.13	61.70
Meropenem	0.0625–64	8	32	38.30	4.26	57.45
Piperacillin	32–512	256	256	0	36.17	63.83
Piperacillin-tazobactam	4–256	32	256	36.17	19.15	44.68
Gentamicin	0.125->1,024	16	>1,024	44.68	2.13	53.19
Amikacin	2->1,024	8	>1,024	59.57	0	40.43
Levofloxacin	0.0625–8	8	8	36.17	10.64	53.19
Ciprofloxacin	0.5–128	64	128	34.04	2.13	63.83
Tetracycline	1–512	512	512	36.17	0	63.83
Doxycycline	0.0625–64	64	64	38.30	0	61.70
Minocycline	0.0625–16	8	16	40.43	25.53	34.04
Trimethoprim-sulfamethoxazole	16->1,024	>1,024	>1,024	40.43	0	59.57
**BIOCIDES**						
Triclosan	2->256	16	128	(53)^b^	(13)^b^	(34)^b^
Chlorhexidine acetate	8–128	16	64	(36)^b^	(17)^b^	(47)^b^
Benzalkonium bromide	4–32	8	32	ND	ND	ND
Sodium hypochlorite	160–640	320	640	ND	ND	ND
Hydrogen peroxide (mM)	47–376	47	94	ND	ND	ND
Ethanol (%, v/v)	7.5–22.5	7.5	15	ND	ND	ND

a *S, susceptible; I, intermediate; R, resistant. ND, not determined (no susceptibility/resistance interpretative breakpoints are available for biocides). The interpretative categories for S, I, and R are based on the CLSI document (CLSI, 2015)*.

b *Values in brackets are based on chlorhexidine or triclosan MIC values of ≤16, 32, ≥64 μg/ml, respectively, for provisional S, I, and R categories (see text for details)*.

**Figure 1:**
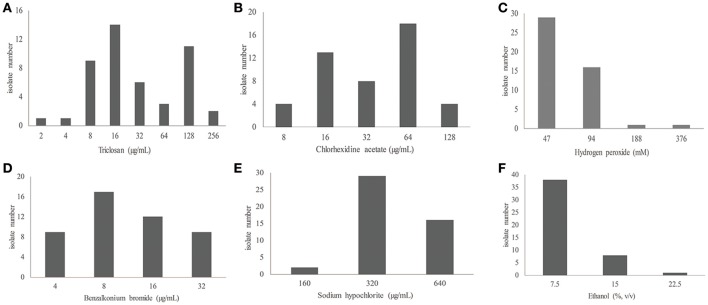
Distribution of the MIC values of biocides for 47 clinical isolates of *A. baumannii*. **(A–F)** are for triclosan, chlorhexidine acetate, hydrogen peroxide, benzalkonium bromide, sodium hypochlorite, and ethanol, respectively.

The tested isolates also showed a variety of susceptibility to biocides. The isolates had MICs ranging from 2 to 256 μg/mL for triclosan, 8 to 128 μg/mL for chlorhexidine, 4–32 μg/mL for benzalkonium bromide, 47–376 mM (1.6–13 mg/mL) for hydrogen peroxide, and 7.5–22.5% (vol/vol) (60–180 mg/mL) for ethanol (Table 2 and Table S3; Figure 1). The MIC values of antibiotics and biocides for the individual isolates are included in Tables S2, S3. Because of the absence of the clinically-relevant interpretative criteria for biocide susceptibility, we could not categorize the isolates as either susceptible or resistant (Table 2). However, the biocide MIC distribution (Figure 1) still allowed for the determination of susceptibility trends of the tested isolates toward biocides. In particular, the bimodal distribution of triclosan and chlorhexidine MIC values clearly indicates the presence of the two subgroups for each of the two biocides (Figure 1). These two subgroups likely represent the wild-type MIC and resistant (or non-susceptible) MIC. In this regard, identification of the wild-type MIC population for bacterial isolates such as the establishment of the microbiologically-based epidemiological cutoff values is critical for resistance surveillance (Turnidge et al., 2006; Martínez et al., 2015). Epidemiological cutoff values would allow for the discrimination of wild-type strains from strains with acquired resistance. Based on the biocide susceptibility results (Figure 1 and Table S3), it is feasible to consider the value of ≤16 μg/ml and ≥64 μg/ml, respectively, as the wild-type MIC and reduced susceptibility (non-susceptible/resistance) cutoff for both triclosan (53 vs. 34%) and chlorhexidine (36 vs. 47%) (Table 2 and Table S3; Figure 1). However, even though the four other biocides tested (benzalkonium, hypochlorite, hydrogen peroxide and ethanol) displayed variations in biocide susceptibility, only one major population can be identified for the isolates for their susceptibility toward the individual agents. Furthermore, these populations likely reflect the intrinsic susceptibility or overlapping wild-type/resistant MICs of *A. baumannii* (Figure 1). Additionally, comparing the absolute MIC values of antibiotics and biocides (Tables S2, S3), biocide agents are less active than antibiotics against susceptible isolates, suggesting a higher intrinsic resistance level of *A. baumannii* isolates to biocides.

The tested isolates are mostly multiple antibiotic resistant (64%; Table S2). However, this resistance phenotype does not clearly link to biocide susceptibility (Tables S2, S3). Moreover, a clear correlation cannot be established for the susceptibility to the six tested biocides (Table S3). In this regard, cross-resistance (reduced susceptibility) to triclosan and chlorhexidine is generally not observed as 13 isolates with reduced triclosan susceptibility (MICs of ≥64 μg/ml) are relatively susceptible to chlorhexidine (MICs of ≤32 μg/ml) while 18 isolates with decreased chlorhexidine susceptibility (MICs of ≥64 μg/ml) are still susceptible to triclosan (MICs of ≤32 μg/ml). It is noted that there were four isolates that had triclosan and chlorhexidine MIC values of ≥64 μg/ml (Table S3).

### Distribution of multidrug resistance transporter genes

Multidrug resistance including resistance to structurally-unrelated antibiotics and biocides can be attributable to broad-specific efflux transporters. Consequently, we assessed the distribution of 11 known drug efflux pump genes, which encode the characterized drug efflux pumps of five different families (i.e., AdeB, AdeG, AdeJ, AdeT1, and AdeT2 of the RND family, AbeD and Amv of the MFS family, AbeM of the MATE family, QacE and QacEΔ1 of the SMR, and AceI of PACE family). Based upon literature information including published genome sequence data, most of these target genes (if not all) are expected to be located on the chromosomes (Li et al., 2015). Indeed, many of the genes were present in the majority of isolates (e.g., 95–100% for *aceI, adeJ, adeT2, abeD, abeM*, and *amvA*). The *adeB* and *adeG* genes were both positive, in 85 and 89% isolates, respectively. Only three genes, *adeT1, qacE*, and *qacE*Δ*1*, were detected at lower prevalence at 75, 70, and 68%, respectively (Table 3).

**Table 3:** Distribution of 12 antimicrobial resistance genes and the MIC values of biocides for 47 clinical isolates of *A. baumannii*^a^.

**Isolate *N* (%)**	**Gene distribution**												**MICs (μg/mL except those specified)**					
	***adeB***	***adeG***	***adeJ***	***adeT1***	***adeT2***	***abeD***	***amvA***	***abeM***	***aceI***	***qacE***	***qacEΔ1***	***fabI***	**TRI**	**CLA**	**BZK**	**SH**	**H_2_O_2_(mM)**	**EtOH (%, v/v)**
22 (47%)	+	+	+	+	+	+	+	+	+	+	+	+	8–128	16–128	8–32	160–640	47–188	7.5–15
4 (8.5%)	+	+	+	+	+	+	+	+	+	-	–	+	8–128	16–64	8–16	320	47	7.5–15
4 (8.5%)	+	+	+	–	+	+	+	+	+	–	–	+	4->256	8–64	4–16	320–640	47–94	7.5–22.5
3 (6.4%)	+	+	+	+	+	+	+	+	+	+	–	+	32–128	8–32	8	320–640	47–94	7.5–15
2 (4.3%)	+	+	+	+	+	+	+	+	+	–	+	+	32–>256	32–64	8–16	640	47–94	7.5
2 (4.3%)	+	+	+	–	+	+	+	+	+	+	+	+	128	16–32	8–16	640	94–376	7.5
1 (2.1%)	+	+	+	+	+	+	–	+	+	+	+	+	64	16	8	640	47	7.5
1 (2.1%)	+	+	+	–	+	+	+	+	+	+	–	+	64	16	4	640	47	7.5
1 (2.1%)	–	+	+	+	+	+	+	+	+	–	+	+	32	8	4	640	94	7.5
1 (2.1%)	–	+	+	–	+	+	+	+	+	+	–	+	2	16	4	320	47	7.5
1 (2.1%)	+	–	+	–	+	+	+	+	+	–	–	+	8	64	8	320	47	7.5
1 (2.1%)	–	+	+	+	+	+	+	+	+	+	+	+	64	8	8	640	94	7.5
1 (2.1%)	–	–	+	+	+	+	+	+	+	–	+	+	32	16	4	640	94	7.5
1 (2.1%)	–	–	+	–	+	+	+	+	+	+	–	+	32	16	4	320	94	7.5
1 (2.1%)	–	–	+	–	–	+	+	–	+	+	+	+	32	16	4	320	47	7.5
1 (2.1%)	–	–	+	–	–	+	+	–	+	–	+	+	16	32	8	640	47	7.5
Percentage of the gene in isolates (*n*)	85% (40/47)	89% (42/47)	100% (47/47)	75% (35/47)	96% (45/47)	100% (47/47)	98% (46/47)	96% (45/47)	100% (47/47)	70% (33/47)	68% (32/47)	100% (47/47)						

a *TRI, triclosan; CLA, chlorhexidine acetate; BZK, benzalkonium bromide; SH, sodium hypochlorite; H_2_O_2_, hydrogen peroxide; EtOH, ethanol. +, positive; –, negative*.

### Presence and mutation of the triclosan target gene *fabI*

Triclosan target gene, *fabI*, was present in all tested isolates. As resistance to triclosan is known to be attributable to specific mutations in *fabI*, DNA sequencing was carried out for PCR-amplified complete *fabI* gene products from 13 highly triclosan resistant isolates (triclosan MICs of ≥128 μg/ml) and 1 triclosan-susceptible isolates (triclosan MICs of 16 μg/ml) (Table S3). Unexpectedly, only one high-level triclosan-resistant isolate (i.e., AB30 [Table S3]) was found to carry a point mutation at amino acid position 95 of FabI, Gly95Ser. Although altered base pairs were observed in *fabI* gene of various isolates, these changes did not alter the amino acid residues of FabI (data not shown).

### Expression of drug resistance transporter genes and their induction by triclosan or chlorhexidine

To investigate the role of efflux pumps in biocide resistance, we targeted the isolates with reduced susceptibility to triclosan and chlorhexidine to analyze gene expression of *adeB, adeJ, adeG, abeM, aceI*, and *fabI*. These genes were targeted based on their known or putative role in mediating antibiotic and/or biocide resistance. The relative expression of *adeB, adeG, adeJ, abeM, aceI*, and *fabI* in comparison with the expression of the housekeeping gene of *16S rRNA* is shown in Table 4. Overall, there was generally detectable, variable expression of these genes which was isolate-specific (i.e., dependent on the gene and isolate). No clear relationship could be established for antibiotic or biocide susceptibility and efflux gene expression levels in individual isolates. However, based on the susceptibility to triclosan, chlorhexidine or antibiotics, the tested isolates (*n* = 24) were divided into three sets of pairs for comparison, i.e., wild-type (WT) (triclosan or chlorhexidine MIC ≤16 μg/ml, or antibiotic susceptible) and non-susceptible (NS) (triclosan or chlorhexidine MIC ≥64 μg/ml, or antibiotic resistant) groups as presented in Table 4. We noted the following gene expression patterns: (i) the relative expression of the *adeB* and *adeJ* genes was doubled or elevated in triclosan non-susceptible isolates compared to the wild-type isolates whereas no significant difference was observed for other four genes (*adeG, abeM, aceI*, and *fabI*); (ii) the relative expression of the *abeM* and *fabI* genes was 2- to 3-fold higher in chlorhexidine non-susceptible isolates compared to the wild-type isolates, while the expression of *adeG* decreased in chlorhexidine non-susceptible isolates; (iii) *aceI* expression in chlorhexidine non-susceptible isolates is 38% higher than that in wild-type isolates; (iv) the relative expression of the *adeJ* and *abeM* genes was 3- to 4-fold higher in antibiotic-resistant isolates than antibiotic-susceptible isolates and there was more than a 2-fold decrease in the *aceI* expression in antibiotic-resistant isolates compared to the susceptible isolates (Table 4).

**Table 4:** The expression of drug resistance transporter genes *adeB, adeG, adeJ, abeM*, and *aceI* and triclosan target gene *fabI* in triclosan- and/or chlorhexidine-resistant isolates *of A. baumannii^a^*.

**Isolate**	**Antibiotic susceptibility^b^**	**MIC (μg/mL)^c^**		**Relative Gene expression**					
		**TRI**	**CLA**	***adeB***	***adeG***	***adeJ***	***abeM***	***aceI***	***fabI***
AB01	S	16	*32*	0.00	0.20	0.17	0.32	1.01	0.26
AB03	MDR	**128**	**64**	0.30	0.08	0.42	0.70	0.29	1.23
AB07	MDR	**128**	16	0.10	1.11	1.08	1.05	0.62	0.76
AB08	MDR	8	**128**	0.23	0.27	0.81	1.27	0.34	0.57
AB09	MDR	8	**128**	0.33	0.30	1.00	1.00	0.29	1.00
AB12	MDR	8	**128**	0.17	0.35	0.70	1.61	0.35	0.33
AB14	S	4	16	0.18	1.08	0.18	0.00	0.94	0.28
AB22	MDR	16	*32*	0.02	0.39	1.30	1.23	0.46	0.14
AB23	S	2	16	0^e^	3.06	0.27	0.00	0.98	0.26
AB24	MDR	**128**	16	0.05	1.00	1.00	1.00	0.25	0.19
AB26	MDR	**128**	16	0.05	0.69	0.67	0.68	0.21	0.12
AB27	MDR	**128**	16	0.05	1.30	0.56	0.71	0.32	0.15
AB30	MDR	>**256**	**64**	1.02	0.29	2.26	4.00	0.52	1.00^d^
AB32	MDR	**128**	*32*	0.26	2.00	3.28	3.28	0.37	1.65
AB33	MDR	8	8	0.02	0.36	0.69	1.16	0.23	1.00
AB34	MDR	**128**	*32*	0.07	0.44	0.88	0.69	0.36	0.69
AB35	S	>**256**	**64**	0.00	1.90	0.62	1.14	2.10	4.37
AB36	MDR	16	**128**	0.13	0.23	0.54	1.01	0.52	0.95
AB37	S	*32*	*32*	0.02	0.60	0.17	0.00	0.78	0.05
AB39	S	**128**	*32*	1.86	0.13	0.13	0.60	1.19	0.24
AB40	MDR	**128**	16	0.03	0.06	1.32	1.32	0.07	0.21
AB42	S	**64**	16	1.01	0.83	0.51	0.61	0.32	0.28
AB43	S	**128**	**64**	0.08	0.14	0.59	0.34	0.19	0.36
AB47	S	**128**	16	0.18	0.05	0.33	0.00	0.29	0.13
All isolates (*n* = 24)		87.6 ± 76.1	46.3 ± 41.1	0.28 ± 0.44	0.70 ± 0.74	0.81 ± 0.70	0.99 ± 0.94	0.54 ± 0.45	0.66 ± 0.91
WT (TRI MIC ≤ 16, *n* = 9)		9.6 ±5.3		0.14 ± 0.12	0.69 ± 0.92	0.62 ± 0.38	0.84 ± 0.59	0.56 ± 0.31	0.53 ± 0.35
NS (TRI MIC ≥ 64, *n* = 14)		141.7 ± 51.3		**0.36** ± **0.55**^f^	0.72 ± 0.67	0.98 ± 0.85	1.15 ± 1.11	0.51 ± 0.53	0.80 ± 1.17
WT (CLA MIC ≤ 16, *n* = 10)			15.1 ± 2.7	0.19 ± 0.30	0.95 ± 0.85	0.66 ± 0.37	0.65 ± 0.50	0.42 ± 0.31	0.34 ± 0.30
NS (CLA MIC ≥ 64, *n* = 8)			96.0 ± 34.2	0.33 ± 0.30	*0.45 ± 0.59*	0.87 ± 0.59	**1.38** ± **1.12**^f^	0.58 ± 0.63	**1.23** ± **1.31**^f^
WT (S to antibiotics, *n* = 9)				0.41 ± 0.65	0.89 ± 1.01	0.35 ± 0.19	0.33 ± 0.39	0.87 ± 0.59	0.69 ± 1.38
NS (R to antibiotics, *n* = 15)				***0.18** ± **0.25***^f^	0.59 ± 0.54	**1.10** ± **0.75**^f^	**1.38** ± **0.97**^f^	***0.35** ± **0.14***^f^	0.67 ± 0.47

a *TRI, triclosan; CLA, chlorhexidine; WT, wild-type; NS, non-susceptible; S, susceptible; R, resistant; MDR, multidrug-resistant*.

b *Antibiotic susceptibility of S and R (MDR) are based on the MIC values from Table S2*.

c *MIC values for non-susceptible (resistant) isolates are shown in bold*.

d *Isolate AB30 carries a Gly95Ser mutation in FabI*.

e *This isolate (AB23) was not detected with adeB by PCR and was excluded in gene expression calculation. There were other isolates (AB01, AB14, AB35, AB37, and AB47) with no detectable gene expression of adeB or abeM where the value “0.00” is given in the table*.

f *The relative gene expression with more than 2-fold change (increase in non-italic and decrease in italic) is shown in bold (except for triclosan non-susceptible isolates, the adeJ expression was increased nearly by 2-fold*.

Investigation was also performed to assess the induction of the above mentioned genes by subinhibitory levels of triclosan or chlorhexidine. Expression of *adeB, adeG, adeJ*, and *fabI* increased about 3-fold upon induction by triclosan while only *adeB* expression was elevated by 4-fold after the induction with chlorhexidine (Table 5).

**Table 5:** Triclosan- or chlorhexidine-induced expression of drug resistance transporter genes *adeB, adeJ, adeG, abeM*, and *aceI* and triclosan target gene *fabI*.

**Isolates**	**Triclosan-induced expression**					**Chlorhexidine-induced expression**				
**(*n* = 24)^a^**	***adeB***	***adeG***	***adeJ***	***abeM***	***fabI***	***adeB***	***adeG***	***adeJ***	***abeM***	***aceI***
Induced	0.93 ± 0.99	2.05 ± 2.72	2.08 ± 3.45	1.52 ± 1.45	2.11 ± 2.25	1.23 ± 2.15	0.99 ± 0.89	1.04 ± 0.82	1.05 ± 1.73	0.72 ± 1.02
Non-induced	0.27 ± 0.44	0.70 ± 0.74	0.81 ± 0.70	0.99 ± 0.94	0.66 ± 0.91	0.27 ± 0.44	0.70 ± 0.74	0.81 ± 0.70	0.99 ± 0.94	0.54 ± 0.45
Gene expression change (fold)^b^	**3.4**	**2.9**	**2.6**	1.5	**3.2**	**4.6**	1.4	1.3	1.1	1.3

a *One isolate (AB23) is absent with adeB and is excluded for adeB gene expression calculation where n = 23*.

b *The relative gene expression with more than 2-fold change after a biocide induction is shown in bold*.

### Genotyping of triclosan-resistant *A. baumannii*

**Figure 2:**
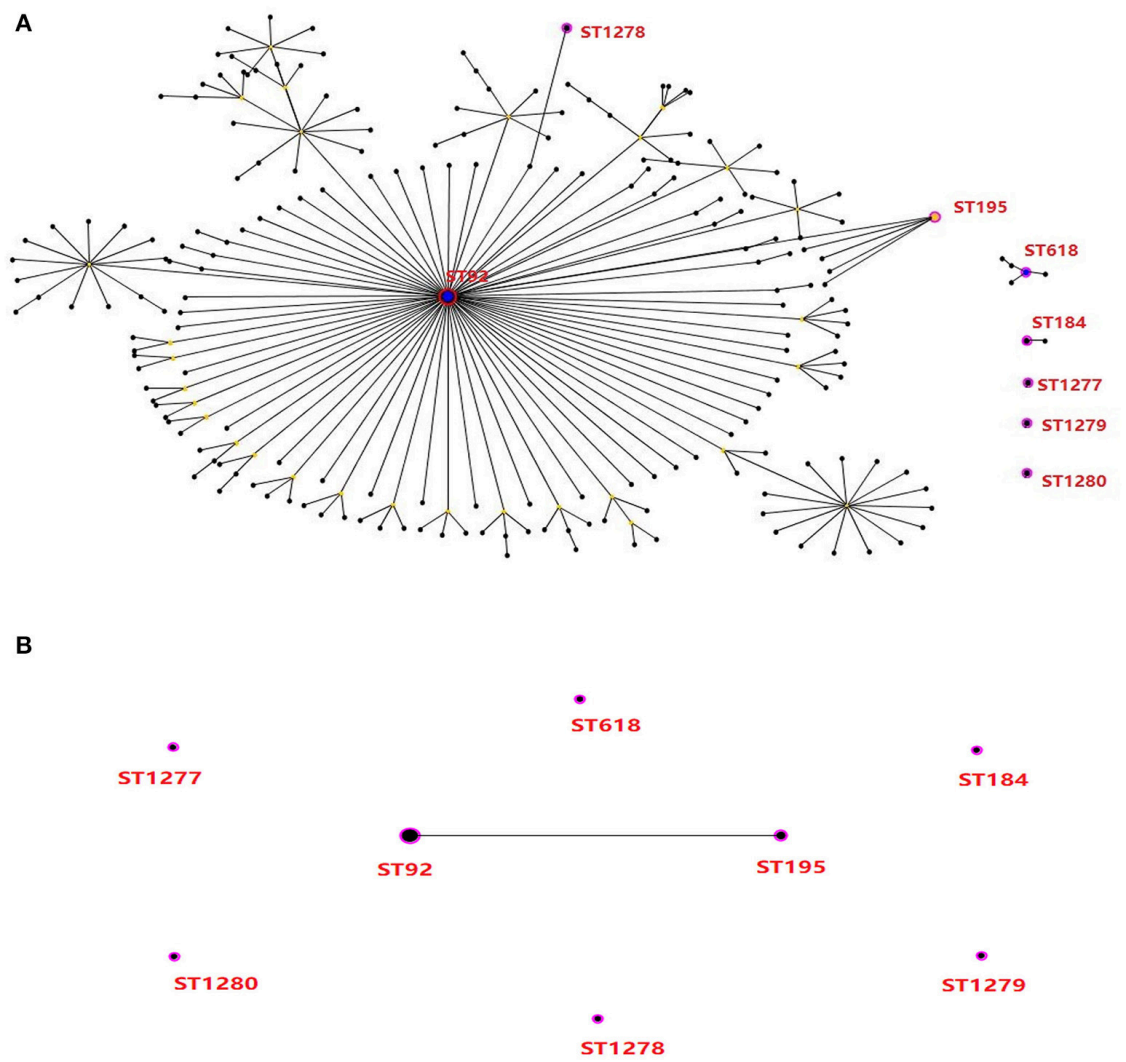
Molecular genotyping of high-level triclosan-resistant clinical isolates of *A. baumannii*. **(A)** Minimum spanning tree analysis by eBURST algorithm of 14 isolates which included 13 isolates with triclosan MICs of 128 μg/mL (8-fold MIC increase in comparison with one relative triclosan-susceptible isolates) based on MLST data. Each circle represents a specific sequence type (ST). The size of each circle homologizes to a different number of isolates, with larger sizes representing higher frequency of occurrence. The solid lines connecting the circles indicate the relationship between different STs. Eight STs showing in red are identified in this study. **(B)** The relationship among the 8 STs for the 14 clinical isolates.

**Figure 3:**
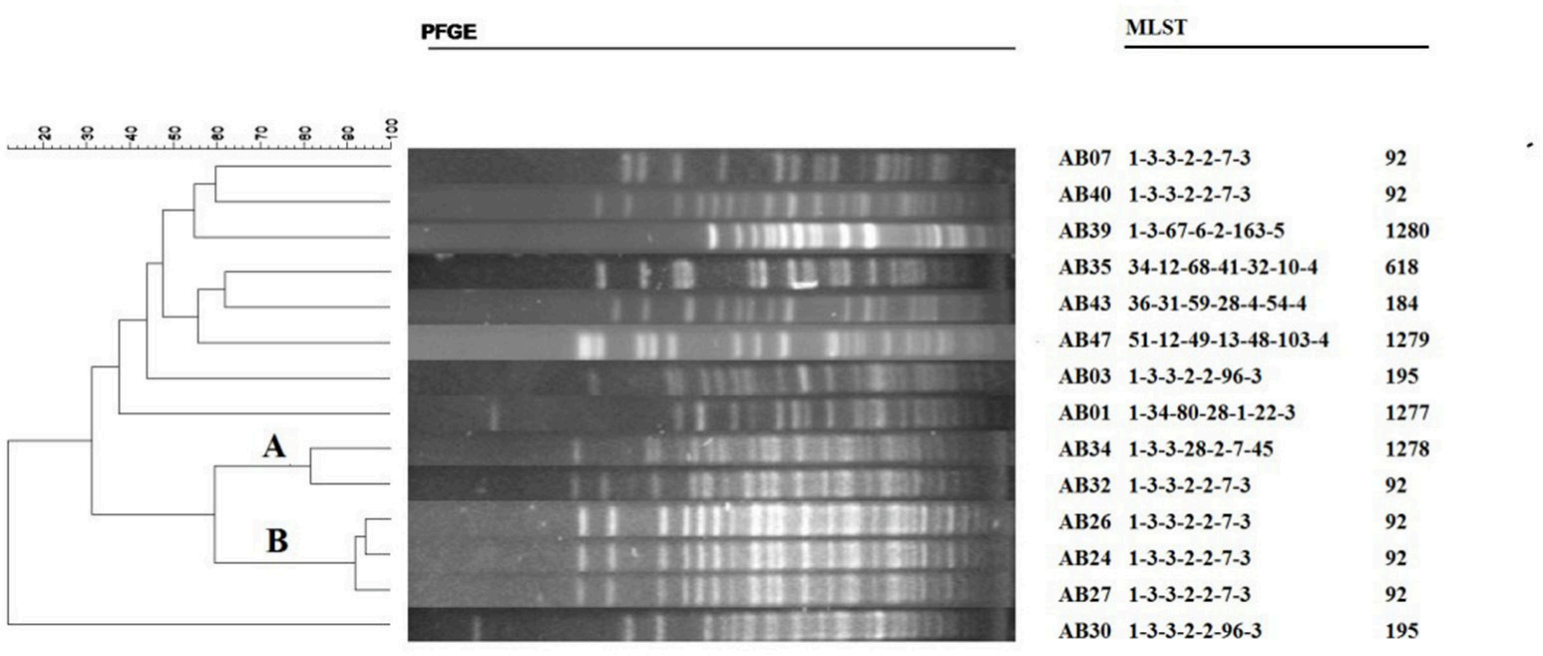
Dendogram illustrating the PFGE patterns of high-level triclosan-resistant clinical isolates of *A. baumannii*. Strain AB01 is triclosan susceptible. The scale bar on the left represents the relatedness by using the percentage. The A and B represent two pulsotype, as the definition of a pulsotype which shows ≥80% relatedness. MLST data on the right shows the specific sequence type (ST).

To investigate the linkage of biocide-resistant *A. baumannii*, the molecular characteristics of 13 isolates with high-level resistance to triclosan (MICs of ≥128 μg/mL; Table S3) were typed by MLST and PFGE. One isolate with triclosan MICs of 16 μg/mL (i.e., isolate AB01) were included as triclosan-susceptible strain for comparison. The MLST analysis revealed a total of 8 different STs (Figures 2, 3), including 4 existing STs (ST92, ST184, ST195, and ST618) and 4 novel STs (ST1277, 1-34-80-28-1-22-3; ST1278, 1-3-3-28-2-7-45; ST1279, 51-12-49-13-48-103-4; ST1280, 1-3-67-6-2-163-5). ST92, accounting for 46.2% (6/13), was the major clone followed by two isolates as ST195 (2/13). The remaining 5 isolates were ST184, ST618, ST1278, ST1279, and ST1280. The triclosan-susceptible isolate belongs to ST1277. Clonal relation analysis showed that ST92 and ST195 belonged to the CC92 lineage, with only one locus (*gpi*) different between the 2 STs, accounting for 61.5% (8/13) of 13 triclosan-resistant isolates. This result suggests that CC92 represents the most widely distributed *A. baumannii* clone complex identified in the hospital for strains with reduced susceptibility to triclosan.

PFGE was analyzed by BioNumerics and >80% homology was used as a standard to distinguish the types. The PFGE profiles contained 14 strains (Figure 3), including pulsotype A (2/13, 15.4%) and pulsotype B (3/13, 23.1%), and the other 7 strains showed different genetic variability pulsotypes. In addition, the MLST results of the clinical isolates showed that CC92 corresponded to the pulsotype B obtained by PFGE.

## Discussion

Antimicrobial resistance in *A. baumannii* is a major threat for the successful treatment and control of infections (Peleg et al., 2008; Ling, 2010). A large number of studies have focused on antibiotic resistance in *A. baumannii* with reduced susceptibility of *A. baumannii* to biocides emerging as a major clinical problem in recent years (Wisplinghoff et al., 2007; Chen et al., 2009; Gnanadhas et al., 2013). The present study clearly shows that there is a high prevalence of multiple antibiotic resistant and biocide resistant isolates in clinical settings. However, our results highlight the challenges in establishing an overall relationship or cross-resistance between antibiotic resistance and reduced susceptibility to biocides. There are at least two possible explanations for our observations. First, the clinical isolates in this study (and others) are not expected to be isogenic but reflect real diverse situations that are faced in clinical settings. Second, importantly, the antibiotics and biocides included in our susceptibility testing exhibit a variety of modes of action that act on different cellular targets, such as cell walls (e.g., β-lactams) proteins (aminoglycosides and tetracyclines), nucleic acids (fluoroquinolones), and fatty acids (triclosan), as well as disruption of membranes (benzalkonium, chlorhexidine, and ethanol) and the generation of oxidative stress (by H_2_O_2_) (McDonnell and Russell, 1999; Li, 2016). Resistance to these agents is mediated by various mechanisms (McDonnell and Russell, 1999; Li, 2016). Indeed, *A. baumannii* genome data reveals the presence of a number of resistance determinants that encode β-lactamases, aminoglycoside-modifying enzymes, tetracycline resistance proteins and broad-spectrum resistance efflux pumps (Fournier et al., 2006; Li, 2016).

Despite the individual resistance mechanisms that explain antimicrobial-specific resistance, multidrug efflux pumps provide a common mechanism of resistance to structurally-related antimicrobial agents and this mechanism is strongly evident in *A. baumannii* (Li et al., 2015; Ling et al., 2016). In this regard, previously suggested outer membrane barrier-related biocide resistance mechanisms could also be attributable to biocide resistance (McDonnell and Russell, 1999; Li et al., 2015). Indeed, Gram-negative bacteria are generally less susceptible to biocides than Gram-positive pathogens (McDonnell and Russell, 1999). Consequently, we assessed the distribution and role of a large number of known or putative drug efflux pumps of several families. Most efflux pump genes are highly prevalent in our tested isolates except for the *qacE* and *qacE*Δ*1* genes. This is likely because the *qacE* and *qacE*Δ*1* genes often exist in the mobile genetic elements such as plasmids and their integration into the chromosome of *A. baumannii* is dependent on specific resistance evolution (Fournier et al., 2006).

Further assessment with qRT-PCR indicates the detectable expression of multiple efflux pump genes, which is consistent with the established roles of drug efflux pumps in intrinsic resistance of *A. baumannii* to multiple antibiotics (Li et al., 2015; Ling et al., 2016). For instance, the most characterized pumps belong to the Ade RND pumps (Coyne et al., 2011; Ling et al., 2016). Gene inactivation studies have demonstrated the contribution of AdeABC, AdeIJK, AbeD, and AmvA pumps to resistance to both antibiotics and biocides (Rajamohan et al., 2010a,b; Srinivasan et al., 2015). Specifically, inactivation of AdeB and AdeJ resulted in, respectively, an 8- and 2-fold reduction in chlorhexidine MIC values (Rajamohan et al., 2010b). AmvA disruption produced a 2- to 4-fold reduction in MIC values to benzalkonium and chlorhexidine (Rajamohan et al., 2010a). AbeD inactivation led to an increased susceptibility to H_2_O_2_, revealing a role of AbeD in oxidative stress tolerance (Srinivasan et al., 2015). Synergistic testing using triclosan/antibiotics and the efflux pump inhibitors also suggests the involvement of efflux pumps in triclosan resistance (Chen et al., 2009).

We focused our study on the mechanisms of resistance to triclosan and chlorhexidine. Triclosan as a bis-phenolic biocide has been widely used in various domestic cleaning products, such as toothpaste, soaps, cosmetics, and hospital equipment (McDonnell and Russell, 1999; Huang et al., 2016; Yueh and Tukey, 2016). One working concentration of triclosan as a hospital disinfectant is 0.003% (30 μg/ml) (Fernández-Cuenca et al., 2015) but we observed a high-level triclosan resistance (triclosan MICs ≥32 μg/mL) in 47% of the tested clinical isolates. This resistance incidence and level are significantly higher than those reported in literature for *A. baumannii* isolates obtained from 2004 to 2005 in China (1.6% [12/732] isolates with MICs ≥4 μg/mL) (Chen et al., 2009). Chlorhexidine is a biguanide disinfectant widely used in health products for infection control [with a working concentration being listed at 4% (McDonnell and Russell, 1999; Fernández-Cuenca et al., 2015)], and consequently, bacterial resistance against chlorhexidine poses a major health risk (Kampf, 2016). Our result of 47% of isolates as chlorhexidine non-susceptible also confirms the resistance problem against chlorhexidine.

We further found that triclosan non-susceptible isolates displayed an increased expression of the *adeB* and *adeJ* genes, while chlorhexidine non-susceptible isolates had the increased expression of *abeM*. Increased expression of the *adeJ* and *abeM* genes was also demonstrated in multiple antibiotic resistant isolates. Moreover, expression of *adeB, adeG*, and/or *adeJ* was further induced by triclosan or chlorhexidine. These results are clearly consistent with the established roles of AdeABC, AdeFGH, AdeIJK, and/or AbeM in antibiotic and biocide resistance. It is currently known that Ade pumps are regulated by two-component systems, AdeSR and BaeSR, and/or repressors, AdeL/AdeN (Ling et al., 2016). These regulatory systems could possibly interact with inducers to derepress the expression of efflux pumps as is demonstrated in the regulation of Gram-negative drug efflux pumps such as in *A. baumannii* and *Pseudomonas aeruginosa* (Li et al., 2015). However, our gene expression data also revealed varied gene expression profiles among the tested isolates. It is possible that these efflux gene expression variations could also partly be strain-specific. For instance, one article described that AdeSR, regulators of AdeABC pump, control multiple gene expression in a strain-specific manner (Richmond et al., 2016).

Additionally, it is important to note that multiple mechanisms are expected to play a role in biocide resistance and the efflux pumps alone cannot entirely explain the high-level biocide resistance (McDonnell and Russell, 1999). Thus, we also targeted the *fabI* gene, which is known to contribute high-level triclosan resistance in Gram-negative bacteria including *A. baumannii* (Heath et al., 1999; Russell, 2004; Chen et al., 2009). Surprisingly, out of 12 high-level triclosan-resistant isolates, only one isolate carried a *fabI* miss–sense mutation (Gly95Ser), although this type of mutation was previously reported in *A. baumannii* and other bacterial species (Tabak et al., 2007; Chen et al., 2009; Fernando et al., 2014; Curiao et al., 2015). This observation suggests possible contribution of additional mechanism(s) rather than *fabI* mutations to triclosan resistance. Indeed, Chen et al. (2009) reported the increased *fabI* expression in low-level triclosan-resistant *A. baumannii* isolates, which aligns with induction of the *fabI* gene expression by triclosan in *Salmonella* spp. (Tabak et al., 2007). Together with our observation for triclosan-induced *fabI* expression in *A. baumannii*, these findings support that triclosan exposure could facilitate the emergence of triclosan resistance via drug target gene overexpression.

The molecular typing results also provide certain insights on epidemiological aspects of high-level triclosan-resistant isolates. The finding of the CC92 clonal isolates as the major clone could suggest that particular clones with reduced biocide susceptibility may spread through clonal expansion in hospitals. Indeed, a recent study also revealed the strong relatedness of extensively-drug-resistant *A. baumannii* to CC92 in hospitals and the natural environment (Seruga Music et al., 2017). Another report also showed the spread of carbapenem-resistant *A. baumannii* belonging to CC92 (Chen et al., 2017); however, biocide susceptibility phenotypes were not assessed. Together with the antibiotic and biocide susceptibility phenotypes of these clones we studied, the present study continues to argue the importance to monitor the biocide susceptibility to *A. baumannii* and to promote biocide stewardship in hospitals (Kampf, 2016; Lanjri et al., 2017).

## Author contributions

FL, XJ, and BL conceived and designed the study. FL, YX, YC, and CL performed the experiments. FL, XJ, and BL analyzed the data and wrote the manuscript. All authors reviewed and approved the final version of the manuscript.

### Conflict of interest statement

The authors declare that the research was conducted in the absence of any commercial or financial relationships that could be construed as a potential conflict of interest.
