# Supplementary material for: Molecular Characterization of Reduced Susceptibility to Biocides in Clinical Isolates of Acinetobacter baumannii
Source: Front Microbiol. 2017 Sep 26;8:1836. doi: 10.3389/fmicb.2017.01836 (PMC5622949; doi:10.3389/fmicb.2017.01836)
Supplement: Supplementary file 1 [file Table1.DOCX]

Supplementary Material

**Molecular Characterization of Reduced Susceptibility to Biocides in Clinical Isolates of *Acinetobacter baumannii***

Fei Lin,^1,2^ Ying Xu,^3^ Yaowen Chang,^1,2^ Chao Liu,^1,2^ Xu Jia,^2^ Baodong Ling,^1^*****

*** Correspondence:**

Baodong Ling

[lingbaodong@cmc.edu.cn](mailto:lingbaodong@cmc.edu.cn)

Supplementary Tables

Table 1S Primers used in this study.

| Gene | Primer | Nucleotide sequence (5’→3’) | Expected size (bp) | Source |
| --- | --- | --- | --- | --- |
| *rpoB* | F | CCTTCATGACCTGGAAYGGNTA | 940 | ([Wang et al., 2014](#_ENREF_8)) |
|  | R | TCCAGGATCTGNCCNACRTTCAT |  |  |
| *16S* | F | CATTATCACGGTAATTAGTG | 216 | ([Chiang et al., 2011](#_ENREF_2)) |
|  | R | AGAGCACTGTGCACTTAAG |  |  |
| *adeB* | F | GAATAAGGCACCGCAACAAT | 124 | ([Nowak et al., 2015](#_ENREF_4)) |
|  | R | TTTCGCAATCAGTTGTTCCA |  |  |
| *adeG* | F | TGAACGATGCTGCTCAAAAC | 681 | ([Nowak et al., 2015](#_ENREF_4)) |
|  | R | CTCCAGCTGTCAACCAGACA |  |  |
| *adeJ* | F | CTTGGTGTAACTGCCGGATT | 605 | ([Nowak et al., 2015](#_ENREF_4)) |
|  | R | TGAGCACCAGACTCACGTTC |  |  |
| *adeT1* | F | TAGAGAATTCGTGTTTGACCCCATTGGTAA | 915 | ([Srinivasan et al., 2011](#_ENREF_7)) |
|  | R | CGGCGGATCCTTATTCATCGTTTAATGCAC |  |  |
| *adeT2* | F | TAGAGAATTCATGGGTAGTACCTCAGGTAT | 717 | ([Srinivasan et al., 2011](#_ENREF_7)) |
|  | R | CGGCGGATCCTTATCTACTCATTGGACATT |  |  |
| *abeD* | F | TTGGCTTGCCAAATGACGTG | 247 | This study |
|  | R | TCTTGACGACTAACCGCACC |  |  |
| *amvA* | F | TGTATTCCTCATTAATATCCC | 989 | ([Rajamohan et al., 2010](#_ENREF_5)) |
|  | R | TTACTTTCTTCGGAAAACTAAACC |  |  |
| *qacE* | F | ATGAAAGGCTGGCTT | 346 | ([Mahzounieh et al., 2014](#_ENREF_3)) |
|  | R | TCACCATGGCGTCGG |  |  |
| *qacEΔ1* | F | ATCGCAATAGTTGGCGAAGT | 226 | This study |
|  | R | CAAGCTTTTGCCCATGAAGC |  |  |
| *fabI* | F | GTGAGATCGGCATGACACAA | 1057 | ([Chen et al., 2009](#_ENREF_1)) |
|  | R | CTGAAGTCCGCTACCGTTAT |  |  |
| *aceI* | F | CCGCGCATTAATCTCGATCTGTACA | 966 | This study |
|  | R | GCAGCTCAGACACCACAACAATAGT |  |  |
| *abeM* | F | AGGGACGTATTATGGCGAAA | 165 | ([Rumbo et al., 2013](#_ENREF_6)) |
|  | R | CTGCTGTGCTTAGACCAATTTTT |  |  |
| *adeB* q-PCR  *adeB* q-PCR | F | AAAGGTATTGGCTACGAGTGG | 132 | This study |
|  | R | TGCCCAGCTTTCATAGAGTG |  |  |
| *adeG* q-PCR  *adeJ* q-PCR | F | GCGTTGCTGTGACAGATGTT | 104 | This study |
|  | R | TTGTGCACGGACCTGATAAA |  |  |
| *adeJ* q-PCR  *adeG* q-PCR | F | CATCGGCTGAAACAGTTGAA | 109 | This study |
|  | R | GCCTGACCATTACCAGCACT |  |  |
| *abeM* q-PCR  *abeM* q-PCR | F | GGTAGGTGTAGGCTTATGGA | 80 | ([Chen et al., 2009](#_ENREF_1)) |
|  | R | CTTCGGCAACTAATGGTGT |  |  |
| *aceI* q-PCR  *aceI* q-PCR | F | CCACCCTCAAAACCAATCGC | 163 | This study |
|  | R | ACATGCCGATGGAAGTGACC |  |  |
| *fabI* q-PCR  *fabI* q-PCR | F | AGGCATAACGCGCTCAGAAC | 175 | ([Chen et al., 2009](#_ENREF_1)) |
|  | R | GCACACGCTTGATGGTGACT |  |  |
| *16S* q-PCR  *16S* q-PCR | F | CAGCTCGTGTCGTGAGATGT | 150 | This study |
|  | R | CGTAAGGGCCATGATGACTT |  |  |

**REFERENCES**

Chen, Y., Pi, B., Zhou, H., Yu, Y., Li, L., (2009). Triclosan resistance in clinical isolates of *Acinetobacter baumannii*. J Med Microbiol 58, 1086-1091. doi:10.1099/jmm.0.008524-0

Chiang, M.C., Kuo, S.C., Chen, Y.C., Lee, Y.T., Chen, T.L., Fung, C.P., (2011). Polymerase chain reaction assay for the detection of Acinetobacter baumannii in endotracheal aspirates from patients in the intensive care unit. J Microbiol Immunol Infect 44, 106-110. doi:10.1016/j.jmii.2010.04.003

Mahzounieh, M., Khoshnood, S., Ebrahimi, A., Habibian, S., Yaghoubian, M., (2014). Detection of antiseptic-resistance genes in *Pseudomonas* and *Acinetobacter* spp. isolated from burn patients. Jundishapur journal of natural pharmaceutical products 9, e15402

Nowak, J., Seifert, H., Higgins, P.G., (2015). Prevalence of eight resistance-nodulation-division efflux pump genes in epidemiologically characterized *Acinetobacter baumannii* of worldwide origin. J Med Microbiol 64, 630-635. doi:10.1099/jmm.0.000069

Rajamohan, G., Srinivasan, V.B., Gebreyes, W.A., (2010). Molecular and functional characterization of a novel efflux pump, AmvA, mediating antimicrobial and disinfectant resistance in *Acinetobacter baumannii*. J Antimicrob Chemother 65, 1919-1925. doi:10.1093/jac/dkq195

Rumbo, C., Gato, E., Lopez, M., Ruiz de Alegria, C., Fernandez-Cuenca, F., Martinez-Martinez, L., Vila, J., Pachon, J., Cisneros, J.M., Rodriguez-Bano, J., Pascual, A., Bou, G., Tomas, M., Spanish Group of Nosocomial, I., Mechanisms of, A., Resistance to, A., Spanish Society of Clinical, M., Infectious, D., Spanish Network for Research in Infectious, D., (2013). Contribution of efflux pumps, porins, and b-lactamases to multidrug resistance in clinical isolates of *Acinetobacter baumannii*. Antimicrob Agents Chemother 57, 5247-5257. doi:10.1128/AAC.00730-13

Srinivasan, V.B., Rajamohan, G., Pancholi, P., Marcon, M., Gebreyes, W.A., (2011). Molecular cloning and functional characterization of two novel membrane fusion proteins in conferring antimicrobial resistance in *Acinetobacter baumannii*. J Antimicrob Chemother 66, 499-504. doi:10.1093/jac/dkq469

Wang, J., Ruan, Z., Feng, Y., Fu, Y., Jiang, Y., Wang, H., Yu, Y., (2014). Species distribution of clinical Acinetobacter isolates revealed by different identification techniques. PLoS One 9, e104882. doi:10.1371/journal.pone.0104882
