# Supplementary material for: Molecular Characterization of Reduced Susceptibility to Biocides in Clinical Isolates of Acinetobacter baumannii
Source: Front Microbiol. 2017 Sep 26;8:1836. doi: 10.3389/fmicb.2017.01836 (PMC5622949; doi:10.3389/fmicb.2017.01836)
Supplement: Supplementary file 2 [file Table2.DOCX]

Supplementary Material

**Molecular Characterization of Reduced Susceptibility to Biocides in Clinical Isolates of *Acinetobacter baumannii***

Fei Lin,^1,2^ Ying Xu,^3^ Yaowen Chang,^1,2^ Chao Liu,^1,2^ Xu Jia,^2^ Baodong Ling,^1^*****

*** Correspondence:**

Baodong Ling

[lingbaodong@cmc.edu.cn](mailto:lingbaodong@cmc.edu.cn)

Supplementary Tables

Table 2S MIC values (in µg/mL) of antibiotics for 47 clinical isolates of *A. baumannii*^a^*.*

| Isolates no | Antibiotic  susceptibility | CAZ | CTX | CRO | IMI | MEM | PIP | TZP | GEN | AMI | LVX | CIP | TET | DOX | MIN | SXT |
| --- | --- | --- | --- | --- | --- | --- | --- | --- | --- | --- | --- | --- | --- | --- | --- | --- |
| AB01 | S | 16 | 4 | 16 | 0.125 | 0.25 | 32 | 8 | 0.5 | 8 | 0.0625 | 1 | 4 | 0.125 | 0.125 | 16 |
| AB02 | S | 16 | 8 | 16 | 0.125 | 0.125 | 32 | 8 | 0.25 | 4 | 0.0625 | 1 | 4 | 0.125 | 0.125 | 16 |
| AB03 | R | 1024 | 128 | 256 | 16 | 8 | 256 | 128 | >1024 | >1024 | 4 | 64 | 256 | 32 | 4 | 32 |
| AB04 | R | 256 | 128 | 256 | 64 | 32 | 256 | 128 | >1024 | >1024 | 4 | 128 | 32 | 1 | 1 | >1024 |
| AB05 | R | 256 | 128 | 256 | 16 | 8 | 256 | 128 | >1024 | >1024 | 8 | 128 | 512 | 64 | 16 | >1024 |
| AB06 | R | 256 | 128 | 256 | 16 | 8 | 256 | 128 | 8 | 8 | 4 | 64 | 512 | 64 | 16 | >1024 |
| AB07 | R | 256 | 128 | 256 | 64 | 32 | 256 | 256 | >1024 | >1024 | 8 | 128 | 512 | 64 | 16 | >1024 |
| AB08 | R | 256 | 128 | 256 | 16 | 8 | 256 | 128 | >1024 | >1024 | 8 | 128 | 512 | 64 | 16 | >1024 |
| AB09 | R | 256 | 128 | 256 | 16 | 8 | 256 | 128 | >1024 | >1024 | 8 | 64 | 512 | 64 | 16 | >1024 |
| AB10 | R | 256 | 128 | 256 | 64 | 32 | 256 | 256 | 16 | 8 | 8 | 128 | 512 | 64 | 8 | >1024 |
| AB11 | R | 256 | 128 | 256 | 64 | 32 | 512 | 256 | >1024 | >1024 | 8 | 128 | 512 | 64 | 8 | >1024 |
| AB12 | R | 256 | 128 | 256 | 64 | 64 | 512 | 256 | >1024 | >1024 | 8 | 64 | 512 | 64 | 16 | >1024 |
| AB13 | R | 256 | 128 | 256 | 64 | 32 | 256 | 256 | >1024 | >1024 | 8 | 128 | 512 | 64 | 16 | >1024 |
| AB14 | S | 16 | 8 | 16 | 0.25 | 0.25 | 32 | 8 | 0.25 | 2 | 0.0625 | 1 | 4 | 0.125 | 0.125 | 32 |
| AB15 | R | 256 | 128 | 256 | 32 | 16 | 256 | 256 | 64 | 8 | 8 | 128 | 512 | 64 | 8 | >1024 |
| AB16 | R | 256 | 128 | 256 | 64 | 32 | 256 | 256 | >1024 | >1024 | 8 | 64 | 512 | 64 | 8 | >1024 |
| AB17 | R | 256 | 128 | 256 | 32 | 16 | 256 | 128 | 128 | 8 | 8 | 128 | 512 | 64 | 8 | >1024 |
| AB18 | R | 256 | 128 | 256 | 64 | 16 | 256 | 256 | 64 | 8 | 8 | 64 | 512 | 64 | 8 | >1024 |
| AB19 | R | 256 | 128 | 256 | 64 | 16 | 256 | 256 | >1024 | >1024 | 8 | 128 | 512 | 64 | 16 | >1024 |
| AB20 | R | 256 | 256 | 256 | 16 | 16 | 256 | 128 | 128 | 8 | 8 | 128 | 512 | 64 | 16 | >1024 |
| AB21 | R | 256 | 256 | 256 | 64 | 64 | 256 | 256 | 2 | 8 | 4 | 128 | 512 | 64 | 8 | 64 |
| AB22 | R | 256 | 256 | 256 | 16 | 16 | 256 | 128 | 2 | 8 | 4 | 64 | 512 | 64 | 8 | >1024 |
| AB23 | S | 16 | 8 | 16 | 0.125 | 0.125 | 32 | 8 | 0.25 | 4 | 0.0625 | 1 | 4 | 0.125 | 0.0625 | 32 |
| AB24 | R | 128 | 64 | 128 | 16 | 8 | 256 | 32 | >1024 | >1024 | 8 | 64 | 512 | 64 | 8 | >1024 |
| AB25 | S | 16 | 4 | 8 | 0.25 | 0.125 | 32 | 8 | 0.25 | 4 | 0.125 | 1 | 4 | 0.125 | 0.125 | 32 |
| AB26 | R | 128 | 64 | 128 | 16 | 8 | 256 | 32 | >1024 | >1024 | 8 | 64 | 512 | 64 | 8 | >1024 |
| AB27 | R | 128 | 64 | 128 | 16 | 4 | 256 | 32 | >1024 | >1024 | 8 | 64 | 512 | 64 | 16 | >1024 |
| AB28 | S | 16 | 8 | 16 | 0.25 | 0.25 | 32 | 8 | 0.5 | 4 | 0.0625 | 1 | 4 | 0.125 | 0.125 | 16 |
| AB29 | S | 16 | 8 | 32 | 0.25 | 0.25 | 64 | 16 | 0.25 | 4 | 0.0625 | 0.5 | 2 | 0.125 | 0.125 | 32 |
| AB30 | R | 1024 | 256 | 512 | 16 | 16 | 256 | 128 | >1024 | >1024 | 8 | 128 | 512 | 64 | 16 | 32 |
| AB31 | S | 16 | 8 | 8 | 0.125 | 0.125 | 32 | 16 | 0.125 | 4 | 0.0625 | 0.5 | 1 | 0.0625 | 0.0625 | 32 |
| AB32 | R | 128 | 64 | 256 | 16 | 8 | 128 | 32 | 4 | 4 | 8 | 64 | 512 | 64 | 16 | >1024 |
| AB33 | R | 256 | 128 | 256 | 64 | 16 | 256 | 128 | 128 | 8 | 8 | 128 | 512 | 64 | 8 | >1024 |
| AB34 | R | 128 | 64 | 256 | 16 | 8 | 128 | 32 | >1024 | >1024 | 8 | 64 | 512 | 64 | 16 | >1024 |
| AB35 | S | 16 | 64 | 8 | 0.25 | 0.125 | 32 | 8 | 0.5 | 8 | 0.0625 | 0.5 | 2 | 0.125 | 0.0625 | 16 |
| AB36 | R | 128 | 64 | 128 | 16 | 4 | 256 | 64 | 4 | 4 | 8 | 64 | 512 | 64 | 16 | >1024 |
| AB37 | S | 16 | 4 | 8 | 0.125 | 0.125 | 32 | 8 | 0.25 | 4 | 0.0625 | 1 | 2 | 0.125 | 0.125 | 32 |
| AB38 | S | 16 | 4 | 16 | 0.25 | 0.25 | 32 | 8 | 0.5 | 4 | 0.0625 | 1 | 2 | 0.25 | 0.125 | 1024 |
| AB39 | S | 16 | 8 | 16 | 0.25 | 0.25 | 32 | 8 | 0.5 | 8 | 0.0625 | 1 | 4 | 0.25 | 0.25 | 32 |
| AB40 | R | 128 | 64 | 128 | 4 | 2 | 128 | 32 | >1024 | >1024 | 8 | 64 | 512 | 64 | 8 | >1024 |
| AB41 | S | 16 | 4 | 8 | 0.0625 | 0.125 | 32 | 8 | 0.5 | 4 | 0.0625 | 1 | 1 | 0.125 | 0.125 | 32 |
| AB42 | S | 16 | 8 | 8 | 0.125 | 0.0625 | 32 | 4 | 0.5 | 4 | 0.0625 | 1 | 2 | 0.125 | 0.0625 | 32 |
| AB43 | S | 16 | 16 | 32 | 0.125 | 0.5 | 64 | 16 | 0.25 | 4 | 0.125 | 2 | 4 | 0.25 | 0.125 | 16 |
| AB44 | R | 128 | 64 | 128 | 16 | 8 | 256 | 32 | >1024 | >1024 | 8 | 64 | 512 | 64 | 16 | >1024 |
| AB45 | S | 16 | 4 | 8 | 0.125 | 0.125 | 32 | 4 | 0.25 | 4 | 0.0625 | 1 | 2 | 0.125 | 0.0625 | 32 |
| AB46 | R | 128 | 64 | 128 | 8 | 8 | 128 | 32 | >1024 | >1024 | 8 | 64 | 512 | 64 | 16 | >1024 |
| AB47 | S | 16 | 4 | 8 | 0.125 | 0.0625 | 32 | 4 | 0.25 | 4 | 0.0625 | 0.5 | 2 | 0.125 | 0.125 | 32 |

^a^CAZ, ceftazidime; CTX, cefotaxime; CRO, ceftriaxone; IMI, imipenem; MEM, meropenem; PIP, piperacillin; TZP, piperacillin-tazobactam; GEN, gentamicin; AMI, amikacin; LVX, levofloxacin; CIP, ciprofloxacin; TET, tetracycline; DOX, doxycycline; MIN, minocycline; SXT, trimethoprim- sulfamethoxazole. S, susceptible; R, resistant.
