# Supplementary material for: Molecular Characterization of Reduced Susceptibility to Biocides in Clinical Isolates of Acinetobacter baumannii
Source: Front Microbiol. 2017 Sep 26;8:1836. doi: 10.3389/fmicb.2017.01836 (PMC5622949; doi:10.3389/fmicb.2017.01836)
Supplement: Supplementary file 3 [file Table3.DOCX]

Supplementary Material

**Molecular Characterization of Reduced Susceptibility to Biocides in Clinical Isolates of *Acinetobacter baumannii***

Fei Lin,^1,2^ Ying Xu,^3^ Yaowen Chang,^1,2^ Chao Liu,^1,2^ Xu Jia,^2^ Baodong Ling,^1^*****

*** Correspondence:**

Baodong Ling

[lingbaodong@cmc.edu.cn](mailto:lingbaodong@cmc.edu.cn)

Supplementary Tables

Table 3S MICs values of biocides and the distribution of drug resistance genes for 47 clinical isolates of *A. baumannii*^a^.

| Isolate | Antibiotic  susceptibility | MIC (µg/ml except those specified) | | | | | | Resistance gene status | | | | | | | | | | | |
| --- | --- | --- | --- | --- | --- | --- | --- | --- | --- | --- | --- | --- | --- | --- | --- | --- | --- | --- | --- |
|  |  | TRI | CLA | BZK | SH | EtOH | H_2_O_2_ | *adeB* | *adeG* | *adeJ* | *adeT1* | *adeT2* | *abeD* | *amvA* | *abeM* | *aceI* | *qacE* | *qacEΔ1* | *fabI* |
| AB01 | S | 16 | 32 | 8 | 320 | 22.5 | 47 | + | + | + | - | + | + | + | + | + | - | - | + |
| AB02 | S | 8 | 64 | 8 | 320 | 15 | 47 | + | - | + | - | + | + | + | + | + | - | - | + |
| AB03 | MDR | 128 | 64 | 16 | 320 | 15 | 47 | + | + | + | + | + | + | + | + | + | - | - | + |
| AB04 | MDR | 16 | 64 | 8 | 320 | 7.5 | 94 | + | + | + | + | + | + | + | + | + | + | + | + |
| AB05 | MDR | 16 | 64 | 16 | 320 | 7.5 | 47 | + | + | + | + | + | + | + | + | + | + | + | + |
| AB06 | MDR | 16 | 64 | 16 | 320 | 7.5 | 47 | + | + | + | + | + | + | + | + | + | + | + | + |
| AB07 | MDR | 128 | 16 | 32 | 160 | 7.5 | 47 | + | + | + | + | + | + | + | + | + | + | + | + |
| AB08 | MDR | 8 | 128 | 16 | 320 | 7.5 | 47 | + | + | + | + | + | + | + | + | + | + | + | + |
| AB09 | MDR | 8 | 128 | 16 | 320 | 7.5 | 47 | + | + | + | + | + | + | + | + | + | + | + | + |
| AB10 | MDR | 16 | 64 | 32 | 320 | 7.5 | 47 | + | + | + | + | + | + | + | + | + | + | + | + |
| AB11 | MDR | 16 | 64 | 16 | 320 | 7.5 | 47 | + | + | + | + | + | + | + | + | + | + | + | + |
| AB12 | MDR | 8 | 128 | 32 | 320 | 7.5 | 47 | + | + | + | + | + | + | + | + | + | + | + | + |
| AB13 | MDR | 8 | 64 | 16 | 320 | 7.5 | 47 | + | + | + | + | + | + | + | + | + | + | + | + |
| AB14 | S | 4 | 16 | 4 | 320 | 15 | 47 | + | + | + | - | + | + | + | + | + | - | - | + |
| AB15 | MDR | 16 | 64 | 32 | 320 | 7.5 | 47 | + | + | + | + | + | + | + | + | + | + | + | + |
| AB16 | MDR | 8 | 64 | 32 | 320 | 7.5 | 47 | + | + | + | + | + | + | + | + | + | + | + | + |
| AB17 | MDR | 8 | 64 | 32 | 160 | 15 | 47 | + | + | + | + | + | + | + | + | + | + | + | + |
| AB18 | MDR | 16 | 64 | 32 | 320 | 15 | 47 | + | + | + | + | + | + | + | + | + | + | + | + |
| AB19 | MDR | 16 | 64 | 32 | 320 | 15 | 47 | + | + | + | + | + | + | + | + | + | + | + | + |
| AB20 | MDR | 16 | 64 | 32 | 320 | 15 | 47 | + | + | + | + | + | + | + | + | + | + | + | + |
| AB21 | MDR | 8 | 64 | 16 | 320 | 15 | 47 | + | + | + | + | + | + | + | + | + | - | - | + |
| AB22 | MDR | 16 | 32 | 16 | 320 | 7.5 | 47 | + | + | + | + | + | + | + | + | + | - | - | + |
| AB23 | S | 2 | 16 | 4 | 320 | 7.5 | 47 | - | + | + | - | + | + | + | + | + | + | - | + |
| AB24 | MDR | 128 | 16 | 8 | 320 | 7.5 | 94 | + | + | + | + | + | + | + | + | + | + | + | + |
| AB25 | S | 32 | 16 | 4 | 320 | 7.5 | 94 | - | - | + | - | + | + | + | + | + | + | - | + |
| AB26 | MDR | 128 | 16 | 8 | 320 | 7.5 | 94 | + | + | + | + | + | + | + | + | + | + | + | + |
| AB27 | MDR | 128 | 16 | 8 | 320 | 7.5 | 94 | + | + | + | + | + | + | + | + | + | + | + | + |
| AB28 | S | 32 | 32 | 8 | 640 | 7.5 | 47 | + | + | + | + | + | + | + | + | + | - | + | + |
| AB29 | S | 32 | 8 | 4 | 640 | 7.5 | 94 | - | + | + | + | + | + | + | + | + | - | + | + |
| AB30 | MDR | >256 | 64 | 16 | 640 | 7.5 | 94 | + | + | + | + | + | + | + | + | + | - | + | + |
| AB31 | S | 32 | 16 | 4 | 320 | 7.5 | 47 | - | - | + | - | - | + | + | - | + | + | + | + |
| AB32 | MDR | 128 | 32 | 8 | 640 | 7.5 | 94 | + | + | + | + | + | + | + | + | + | + | + | + |
| AB33 | MDR | 8 | 8 | 4 | 640 | 7.5 | 188 | + | + | + | + | + | + | + | + | + | + | + | + |
| AB34 | MDR | 128 | 32 | 8 | 640 | 7.5 | 376 | + | + | + | - | + | + | + | + | + | + | + | + |
| AB35 | S | >256 | 64 | 16 | 640 | 7.5 | 94 | + | + | + | - | + | + | + | + | + | - | - | + |
| AB36 | MDR | 16 | 128 | 16 | 640 | 7.5 | 94 | + | + | + | - | + | + | + | + | + | + | + | + |
| AB37 | S | 32 | 32 | 8 | 640 | 7.5 | 47 | + | + | + | + | + | + | + | + | + | + | - | + |
| AB38 | S | 32 | 16 | 4 | 640 | 7.5 | 94 | - | - | + | + | + | + | + | + | + | - | + | + |
| AB39 | S | 128 | 32 | 8 | 640 | 7.5 | 94 | + | + | + | + | + | + | + | + | + | + | - | + |
| AB40 | MDR | 128 | 16 | 8 | 320 | 7.5 | 94 | + | + | + | + | + | + | + | + | + | + | + | + |
| AB41 | S | 16 | 32 | 8 | 640 | 7.5 | 47 | - | - | + | - | - | + | + | - | + | - | + | + |
| AB42 | S | 64 | 16 | 4 | 640 | 7.5 | 47 | + | + | + | - | + | + | + | + | + | + | - | + |
| AB43 | S | 128 | 64 | 8 | 320 | 7.5 | 47 | + | + | + | + | + | + | + | + | + | - | - | + |
| AB44 | MDR | 64 | 16 | 8 | 640 | 7.5 | 94 | + | + | + | + | + | + | - | + | + | + | + | + |
| AB45 | S | 16 | 8 | 4 | 640 | 7.5 | 94 | + | + | + | - | + | + | + | + | + | - | - | + |
| AB46 | MDR | 64 | 8 | 8 | 640 | 7.5 | 94 | - | + | + | + | + | + | + | + | + | + | + | + |
| AB47 | S | 128 | 16 | 8 | 320 | 7.5 | 47 | + | + | + | + | + | + | + | + | + | + | - | + |
| Percentage of the gene in the isolates | | | | | | | | 85%  (40/47) | 89%  (42/47) | 100%  (47/47) | 75%  (35/47) | 96%  (45/47) | 100%  (47/47) | 100%  (47/47) | 96%  (45/47) | 100%  (47/47) | 70%  (33/47) | 68%  (32/47) | 100%  (47/47) |

^a^TRI, triclosan; CLA, chlorhexidine acetate; BZK, benzalkonium bromide; SH, sodium hypochlorite; EtOH, ethanol; H_2_O_2_, hydrogen peroxide; +, positive; -, negative; S, susceptible; MDR, multidrug resistant; MICs for ethanol in % (v/v) and for hydrogen peroxide in mM
